# Evaluation of the Efficacy & Biochemical Mechanism of Cell Death Induction by *Piper longum* Extract Selectively in *In-Vitro* and *In-Vivo* Models of Human Cancer Cells

**DOI:** 10.1371/journal.pone.0113250

**Published:** 2014-11-17

**Authors:** Pamela Ovadje, Dennis Ma, Phillip Tremblay, Alessia Roma, Matthew Steckle, Jose-Antonio Guerrero, John Thor Arnason, Siyaram Pandey

**Affiliations:** 1 Department of Chemistry & Biochemistry, University of Windsor, Windsor, ON, Canada; 2 Department of Biology, University of Ottawa, Ottawa, ON, Canada; Texas Tech University Health Sciences Center, United States of America

## Abstract

**Background:**

Currently chemotherapy is limited mostly to genotoxic drugs that are associated with severe side effects due to non-selective targeting of normal tissue. Natural products play a significant role in the development of most chemotherapeutic agents, with 74.8% of all available chemotherapy being derived from natural products.

**Objective:**

To scientifically assess and validate the anticancer potential of an ethanolic extract of the fruit of the Long pepper (PLX), a plant of the *piperaceae* family that has been used in traditional medicine, especially Ayurveda and investigate the anticancer mechanism of action of PLX against cancer cells.

**Materials & Methods:**

Following treatment with ethanolic long pepper extract, cell viability was assessed using a water-soluble tetrazolium salt; apoptosis induction was observed following nuclear staining by Hoechst, binding of annexin V to the externalized phosphatidyl serine and phase contrast microscopy. Image-based cytometry was used to detect the effect of long pepper extract on the production of reactive oxygen species and the dissipation of the mitochondrial membrane potential following Tetramethylrhodamine or 5,5,6,6′-tetrachloro-1,1′,3,3′-tetraethylbenzimidazolylcarbocyanine chloride staining (JC-1). Assessment of PLX *in-vivo* was carried out using Balb/C mice (toxicity) and CD-1 nu/nu immunocompromised mice (efficacy). HPLC analysis enabled detection of some primary compounds present within our long pepper extract.

**Results:**

Our results indicated that an ethanolic long pepper extract selectively induces caspase-independent apoptosis in cancer cells, without affecting non-cancerous cells, by targeting the mitochondria, leading to dissipation of the mitochondrial membrane potential and increase in ROS production. Release of the AIF and endonuclease G from isolated mitochondria confirms the mitochondria as a potential target of long pepper. The efficacy of PLX in *in-vivo* studies indicates that oral administration is able to halt the growth of colon cancer tumors in immunocompromised mice, with no associated toxicity. These results demonstrate the potentially safe and non-toxic alternative that is long pepper extract for cancer therapy.

## Introduction

The continuing increase in the incidence of cancer signifies a need for further research into more effective and less toxic alternatives to current treatments. In Canada alone, it was estimated that 267,700 new cases of cancer will arise, with 76,020 deaths occurring in 2012 alone. The global statistics are even more dire, with 12.7 million cancer cases and 7.6 million cancer deaths arising in 2008 [1], [2]. The hallmarks of cancer cells uncover the difficulty in targeting cancer cells selectively. Cancer cells are notorious for sustaining proliferative signaling, evading growth suppression, activating invasion and metastasis and resisting cell death among other characteristics [3]. These characteristics pose various challenges in the development of successful anticancer therapies. The ability of cancer cells to evade cell death events has been the center of attention of much research, with focus centered on targeting the various vulnerable aspects of cancer cells to induce different forms of Programmed Cell Death (PCD) in cancer cells, with no associated toxicities to non-cancerous cells.

Apoptosis (PCD type I) has been studied for decades, the understanding of which will enhance the possible development of more effective cancer therapies. This is a form of cell death that is required for regular cell development and homeostasis, as well as a defense mechanism to get rid of damaged cells; cells undergoing apoptosis invest energy in their own demise so as not to become a nuisance [2]. Cancer cells evade apoptosis in order to confer added growth advantage and sustenance, therefore current anticancer therapies endeavour to exploit the various vulnerabilities of cancer cells in order to trigger the activation of apoptosis through either the extrinsic or intrinsic pathways [4], [5]. The challenges facing some of the available cancer therapies are their abilities to induce apoptosis in cancer cells by inducing genomic DNA damage. Although this is initially effective, as they target rapidly dividing cells [6], they are usually accompanied by severe side effects caused by the non-selective targeting of normal non-cancerous cells, suggesting a need for other non-common targets for apoptosis induction without the associated toxicities.

Natural health products (NHPs) have shown great promise in the field of cancer research. The past 70 years have introduced various natural products as the source of many drugs in cancer therapy. Approximately 75% of the approved anticancer therapies have been derived from natural products, an expected statistic considering that more than 80% of the developing world's population is dependent on the natural products for therapy [7]. Plant products especially contain many bioactive chemicals that are able to play specific roles in the treatment of various diseases. Considering the complex mixtures and pharmacological properties of many natural products, it becomes difficult to establish a specific target and mechanism of action of many NHPs. With NHPs gaining momentum, especially in the field of cancer research, there is a lot of new studies on the mechanistic efficacy and safety of NHPs as potential anticancer agents [8].

Long pepper, from the Piperaceae family, has been used for centuries for the treatment of various diseases. Several species of long pepper have been identified, including *Piper longum* (the extract of which is being used in this study), *Piper betle*, *Piper retrofactum*, extracts of which have been used for years in the treatment of various diseases. A long list of uses and benefits are associated with extracts of different *Piper spp*, with reports indicating their effectiveness as good digestive agents and pain and inflammatory suppressants [9]. However, there is little to no scientific validation, only anecdotal evidence, for the benefits associated with the use of long pepper extracts. There are scientific studies have been carried out on several compounds present in extracts of long pepper, including piperines, which has been shown to inhibit many enzymatic drug bio-transforming reactions and plays specific roles in metabolic activation of carcinogens and mitochondrial energy production [10]–[13], and various piperidine alkaloids, with fungicidal activity [9], [14]. Some of these compounds have shown potent anticancer activity [15], suggesting that Long pepper extracts could represent a new NHP, with better selective efficacy against cancer cells.

In this study, we examine the efficacy of an ethanolic extract of Long pepper fruit (PLX) against various cancer cells, as well as attempt to elucidate the mechanism of action, following treatment. Results from this study demonstrate that PLX reduced the viability of various cancer cell types in a dose and time dependent manner, where apoptosis induction was observed, following mitochondrial targeting and the release of pro-apoptotic factors. Due to the low doses of PLX required to induce apoptosis in cancer cell, it was easy to find the therapeutic window of this extract. The induction of apoptosis was found to be caspase-independent, although there was activation of both the extrinsic and intrinsic pathways and the production of ROS was not essential to the mechanism of cell death induction by PLX. The complex polychemical extract of the fruit of the long pepper plant, as a natural health product with unprecedented anticancer activity, provides a way to target multiple vulnerabilities of cancer cells. Even in the presence of certain inhibitors, PLX was efficacious in inducing apoptosis suggesting the potential application of developing PLX as a safe and efficacious cancer therapy.

## Materials and Methods

Animal studies were carried out according to the animal care committee protocol approved by the University of Windsor Animal Care Committee; This protocol and project was approved by the animal care committee – Protocol number: AUPP 10–17), in accordance with the Canadian Council of Animal Care (CCAC) guidelines.

### Cell Culture

The malignant melanoma cell line G-361, human colorectal cancer cell lines HT-29 and HCT116 (American Type Culture Collection, Manassas, VA, USA Cat. No. CRL-1687, CCL-218 & CCL-247, respectively) were cultured with McCoy's Medium 5a (Gibco BRL, VWR, Mississauga, ON, Canada) supplemented with 10% (v/v) FBS (Thermo Scientific, Waltham, MA, USA) and 40 mg/ml gentamicin (Gibco, BRL, VWR). The ovarian adenocarcinoma cell line OVCAR-3 (American Type Culture Collection, Cat. No. HTB-161) was cultured in RPMI-1640 media (Sigma-Aldrich Canada, Mississauga, ON, Canada) supplemented with 0.01 mg/mL bovine insulin, 20% (v/v) fetal bovine serum (FBS) standard (Thermo Scientific, Waltham, MA, USA) and 10 mg/mL gentamicin. The pancreatic adenocarcinoma cell line BxPC-3 (American Type Culture Collection, Cat. No. CRL-1424) was cultured in RPMI-1640 medium, supplemented with 10% (v/v) fetal bovine serum (FBS) standard and 40 mg/mL gentamicin. Normal-derived colon mucosa NCM460 cell line (INCELL Corporation, LLC., San Antonio, TX, USA) was grown in INCELL's M3Base medium (INCELL Corporation, LLC., Cat. No. M300A500) supplemented with 10% (v/v) FBS and 10 mg/mL gentamicin.

All cells were grown in optimal growth conditions of 37°C and 5% CO2. Furthermore, all cells were passaged for ≤6 months.

### Long Pepper Extraction

Ripe and dried Indian long pepper fruits were obtained from Quality Natural Foods limited, Toronto Ontario. The plant material was ground up and extracted in anhydrous ethanol (100%) in a ratio of 1∶10 (1 g plant material to 10 ml ethanol). The extraction was carried out overnight on a shaker at room temperature. The extract was passed through a P8 coarse filter, followed by a 0.45 µm filter. The solvent was evaporated using a RotorVap at 40°C and reconstituted in dimethylsulfoxide (Me_2_SO) at a final stock concentration of 450 mg/ml.

### Cell Treatment

Cells were plated and grown to 60–70% confluence, before being treated with Long Pepper Extracts (PLX), N-Acetyl-L-cysteine (NAC) (Sigma-Aldrich Canada, Cat. No. A7250), and broad-spectrum caspase inhibitor, Z-VAD-FMK (EMD Chemicals, Gibbstown, NJ, USA) at the indicated doses and durations. NAC was dissolved in sterile water. Z-VAD-FMK was dissolved in dimethylsulfoxide (Me_2_SO). PLX was extracted as previously described, reconstituted in Me_2_SO. Before treatment, a dilute working concentration of 10 mg/ml in PBS was prepared. Cells were treated with the 10 mg/ml to obtained the final concentrations indicated in the results section.

### Assessing the Efficacy of Long Pepper Extract (PLX) In Cancer Cells

#### WST-1 Assay for Cell Viability

To assess the effect of PLX on cancer cells, a water-soluble tetrazolium salt (WST-1) based colorimetric assay was carried out as per manufacturer's protocol (Roche Applied Science, Indianapolis, IN, USA), to quantify cell viability as a function of cellular metabolism. Equal number of cells were seeded onto 96-well clear bottom tissue culture plates then treated with the indicated treatments at the indicated concentrations and durations. Following treatment, cells were incubated with the WST-1 reagent for 4 hours at 37°C with 5% CO2. The WST-1 reagent is cleaved to formazan by cellular enzymes in actively metabolizing cells. The formazan product was quantified by taking absorbance readings at 450 nm on a Wallac Victor^3^ 1420 Multilabel Counter (PerkinElmer, Woodbridge, ON, Canada). Cellular viability as a measure of metabolic activity was expressed as percentages of the solvent control groups.

#### Nuclear Staining

Subsequent to treatment, the nuclei of cells were stained with 10 µM Hoechst 33342 dye (Molecular Probes, Eugene, OR, USA) or 1 mg/ml propidium iodide (PI) (Sigma Aldrich, Mississauga, ON. Canada), to monitor nuclear morphology for apoptosis induction at designated time points and overall cell death. Cells were incubated with 10 µM Hoechst dye and 1 mg/ml PI for 10 minutes and micrographs were taken with a Leica DM IRB inverted fluorescence microscope (Wetzlar, Germany) at 400× magnification. Image-based cytometry was used to quantify the amount of cell death occurring with PI staining.

#### Annexin V Binding Assay

To confirm the induction of apoptosis, the binding of Annexin V to externalized phosphatidylserine on the outer cellular surface, was assessed. Following treatment with PLX, cells were washed twice in phosphate buffer saline (PBS). Subsequently, cells were resuspended and incubated in Annexin V binding buffer (10 mM HEPES, 10 mM NaOH, 140 mM NaCl, 1 mM CaCl2, pH 7.6) with Annexin V AlexaFluor-488 (1∶50) (Invitrogen, Canada, Cat No. A13201) for 15 minutes. In the final 10 minutes of incubation, 10 µM Hoechst and 1 mg/ml propidium iodide were added to the microcentrifuge tube and incubated for the final 10 minutes in the dark. Micrographs were taken at 400× magnification on a Leica DM IRB inverted microscope (Wetzlar, Germany) and image-based cytometry was used to quantify the percentage of programmed cell death (annexin V positive cells) occurring after treatment.

#### TUNEL Staining to Detect DNA Damage and Quantify Apoptosis

Following PLX treatment, HT-29 cells were labeled with the Terminal deoxynucleotidyl transferase dUTP nick end labeling (TUNEL) assay. The assay was performed according to the manufacturer's protocol (Molecular Probes, Eugene, OR), in order to detect DNA damage. Cells were treated with PLX or VP-16 (as a positive control) at indicated concentrations and time points and analyzed for the fragmentation of DNA. Following treatment, cells were fixed by suspending them in 70% (v/v) ethanol and stored at −20°C overnight. The sample was then incubated with a DNA labeling solution (10 µL reaction buffer, 0.75 µL TdT enzyme, 8 µL BrdUTP, 31.25 µL of dH2O) for 1 hour at 25°C. Each sample was exposed to an antibody solution (5 µL Alexa Fluor 488 labeled anti-BrdU antibody and 95 µL rinse solution). The cells were incubated with the antibody solution for 20 minutes and TUNEL positive cells were quantified by image-based cytometry.

#### Whole Cell ROS Generation

Following treatment with PLX, cells were incubated with 2′,7′-Dichlorofluorescin diacetate H_2_DCFDA (Catalog No. D6883, Sigma Aldrich, Mississauga ON. Canada) for 45 minutes. Cells were collected, washed twice in PBS and green fluorescence was observed using a TALI image-based cytometer (Invitrogen, Canada). NAC was used to assess the dependence of PLX on ROS generation and viability.

### Assessment of Mitochondrial Function Following PLX Treatment

#### Tetramethylrhodamine Methyl Ester (TMRM) Staining

To monitor mitochondrial membrane potential (MMP), tetramethylrhodamine methyl ester (TMRM) (Gibco BRL, VWR, Mississauga, ON, Canada) or 5,5,6,6′-tetrachloro-1,1′,3,3′-tetraethylbenzimidazolylcarbocyanine chloride (JC-1) (Invitrogen, Canada) were used. Cells were grown on coverslips, treated with the indicated concentrations of treatments at the indicated time points, and incubated with 200 nM TMRM for 45 minutes at 37°C. Micrographs were obtained at 400× magnification on a Leica DM IRB inverted fluorescence microscope (Wetzlar, Germany). To confirm the results obtained by fluorescence microscopy, image-based cytometry was used to detect red fluorescence. Cells were seeded in 6-well plates and following treatment, cells were incubated with TMRM for 45 minutes, washed twice in PBS and placed in TALI slides. Red fluorescence was obtained using a TALI image-based cytometer (Invitrogen, Canada).

#### Mitochondrial Isolation to Assess Mitochondrial Targeting

Cells were collected by trypsin, washed once in cold PBS, resuspended in cold hypotonic buffer (1 mM EDTA, 5 mM Tris–HCl, 210 mM mannitol, 70 mM sucrose, 10 µM Leu-pep and Pep-A, 100 µM PMSF), and manually homogenized. The homogenized cell solution was centrifuged at 3000 rpm for 5 minutes at 4°C. The supernatant was centrifuged at 12,000 rpm for 15 minutes at 4°C and the mitochondrial pellet was resuspended in cold reaction buffer (2.5 mM malate, 10 mM succinate, 10 µM Leu-pep and Pep-A, 100 µM PMSF in PBS). The isolated mitochondria were treated with PLX at the indicated concentrations and incubated for 2 hours in cold reaction buffer. The control group was treated with solvent (ethanol). Following 2 hour incubation with extract, mitochondrial samples were vortexed and centrifuged at 12,000 rpm for 15 minutes at 4°C. The resulting supernatant and mitochondrial pellets (resuspended in cold reaction buffer) were subjected to Western Blot analysis to assess for the mitochondrial release/retention of pro-apoptotic factors.

#### Western Blot Analyses

Protein samples were subjected to SDS-PAGE, transferred onto a nitrocellulose membrane, and blocked with 5% w/v milk TBST (Tris-Buffered Saline Tween-20) solution for 1 hour. Membranes were incubated overnight at 4°C with an anti-endonuclease G (EndoG) antibody (1∶1000) raised in rabbits (Abcam, Cat. No. ab9647, Cambridge, MA, USA), an anti-succinate dehydrogenase subunit A (SDHA) antibody (1∶1000) raised in mice (Santa Cruz Biotechnology, Inc., sc-59687, Paso Robles, CA, USA), or an anti-apoptosis inducing factor (AIF) antibody raised in rabbits (1∶1000) (Abcam, Cat. No. ab1998, Cambridge, MA, USA). After primary antibody incubation, the membrane was washed once for 15 minutes and twice for 5 minutes in TBST. Membranes were incubated for 1 hour at room temperature with an anti-mouse or an anti-rabbit horseradish peroxidase-conjugated secondary antibody (1∶2000) (Abcam, ab6728, ab6802, Cambridge, MA, USA) followed by three 5-minute washes in TBST. Chemiluminescence reagent (Sigma-Aldrich, CPS160, Mississauga, ON, Canada) was used to visualize protein bands and densitometry analysis was performed using ImageJ software.

### *In-Vivo* Assessment of Long Pepper Extract

#### Toxicity Assessment

Six week old Balb/C mice were obtained from Charles River Laboratories and housed in constant laboratory conditions of a 12-hour light/dark cycle, in accordance with the animal protocols outlined in the University of Windsor Research Ethics Board- AUPP 10–17). Following acclimatization, mice were divided into three groups (3 animals/control (untreated), 3 animals/gavage control (vehicle treatment) and 4 animals/treatment group). The control untreated group was given plain filtered water, while the second and third group was given 50 mg/kg/day vehicle (Me_2_SO) or PLX, respectively for 75 days. During the period of study, toxicity was measured by weighing mice twice a week and urine was collected for protein urinalysis by urine dipstick and Bradford assays. Following the duration of study, mice were sacrificed and their organs (livers, kidneys and hearts) were obtained for immunohistochemical and toxicological analysis by Dr. Brooke at the University of Guelph.

#### Efficacy of PLX in Tumor Xenograft Models of Immunocompromised Mice

Six week old male CD-1 nu/nu mice were obtained from Charles River Laboratories and housed in constant laboratory conditions of a 12-hour light/dark cycle, in accordance with the animal protocols outlined in the University of Windsor Research Ethics Board- AUPP 10–17). Following acclimatization, the mice were injected subcutaneously in the right and left hind flanks with a colon cancer cell suspension (in Phosphate buffered saline) at a concentration of 2 * 10^6^ cells/mouse (HT-29, p53^−/−^, in the left flank and HCT116, p53^+/+^, in the right flank). Tumors were allowed to develop (approximately a week), following which the animals were randomized into treatment groups of 4 mice per group, a control group, a gavage control group given plain filtered sterile water, as well as gavage regimen of the vehicle (5 µL Me_2_SO in PBS) twice a week. The final group was given filtered water supplemented with long pepper extract at a concentration of 100 µg/mL, as well as gavage regimen of long pepper extract (5 µL extract in PBS), twice a week, corresponding to 50 mg/kg/day. The tumors were assessed every other day by measuring the length, width and height, using a standard caliper and the tumor volume was calculated according to the formula π/6*length*width. The mice were also assessed for any weight loss every other day for the duration of the study, which lasted 75 days, following which the animals were sacrificed and their organs and tissues (liver, kidneys, heart and tumors) were obtained and stored in 10% formaldehyde for immunohistochemical and toxicological analysis.

#### Hematoxylin & Eosin (H & E) Staining

Mice organs were fixed in 10% formaldehyde, following which they were cryosectioned into 10 µm sections and placed on a superfrost/Plus microscope slides (Fisherbrand, Fisher Scientific). Sections of organs were stained according to a standardized H & E protocol [16].

### Phytochemical Analysis of Long Pepper Extract by HPLC

HPLC analysis of the long pepper crude extract was carried out at University of Ottawa in the Arnason lab. A total of five well-known piperamides were analyzed and compared to the crude long pepper extract. The extracts and piperamide standards were analyzed on a Luna C18-5u-250×4.6 mm column at 45°C at a flow rate of 1.0 mL/min with a mobile phase constituted of H_2_O and methanol as outlined in Table 1. Chromatogram profiles were used to detect the any differences between a sample standard of known piperamides in the crude long pepper extracts.

**Table 1:** Analysis of five well-known piperamides and crude long pepper extract at a flow rate of 1.0 mL/min with a mobile phase constituted of H_2_O and methanol.

Time (mins)	H_2_0 (%)	MeOH (%)
**0.0**	37.5	62.5
**15.0**	35.0	65.0
**35.0**	0.0	100.0
**45.0**	0.0	100.0
**46.0**	37.5	62.5

### Statistical Analysis

All experiments were repeated at least three independent times. Representative fluorescence images were shown, where appropriate. Statistical analysis was performed using GraphPad Prism 6.0 288 software. The mean and standard error of three independent experiments were analyzed for the quantification data. The Student's T-test and two-way Anova were used for statistical analysis.

## Results

### Ethanolic Extract of Long Pepper (PLX) Effectively and Selectively Reduces the Viability of & Induces Apoptosis in Cancer cells in a Dose & Time Dependent Manner

**Figure 1:**
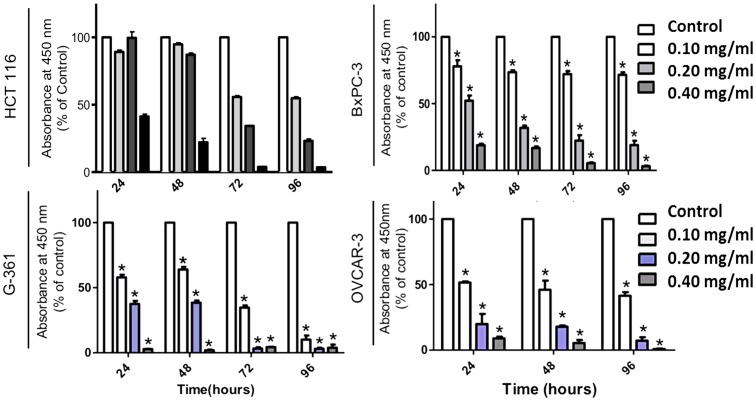
Crude Ethanolic Extract of Long Pepper (PLX) Effectively Reduces the Percentage of Viable Cancer cells in a Dose & Time Dependent Manner. Colon (HCT116), Ovarian (OVCAR-3), Pancreatic (BxPC-3) cancer and Melanoma (G-361) cells were treated with a crude ethanolic extract of long pepper (PLX), following which they were incubated with WST-1 cell viability dye for 4 hours. Absorbance was read at 450 nm and expressed as a percent of the control. Values are expressed as mean ± SD from quadruplicates of 3 independent experiments. **P<0.0001.

**Figure 2:**
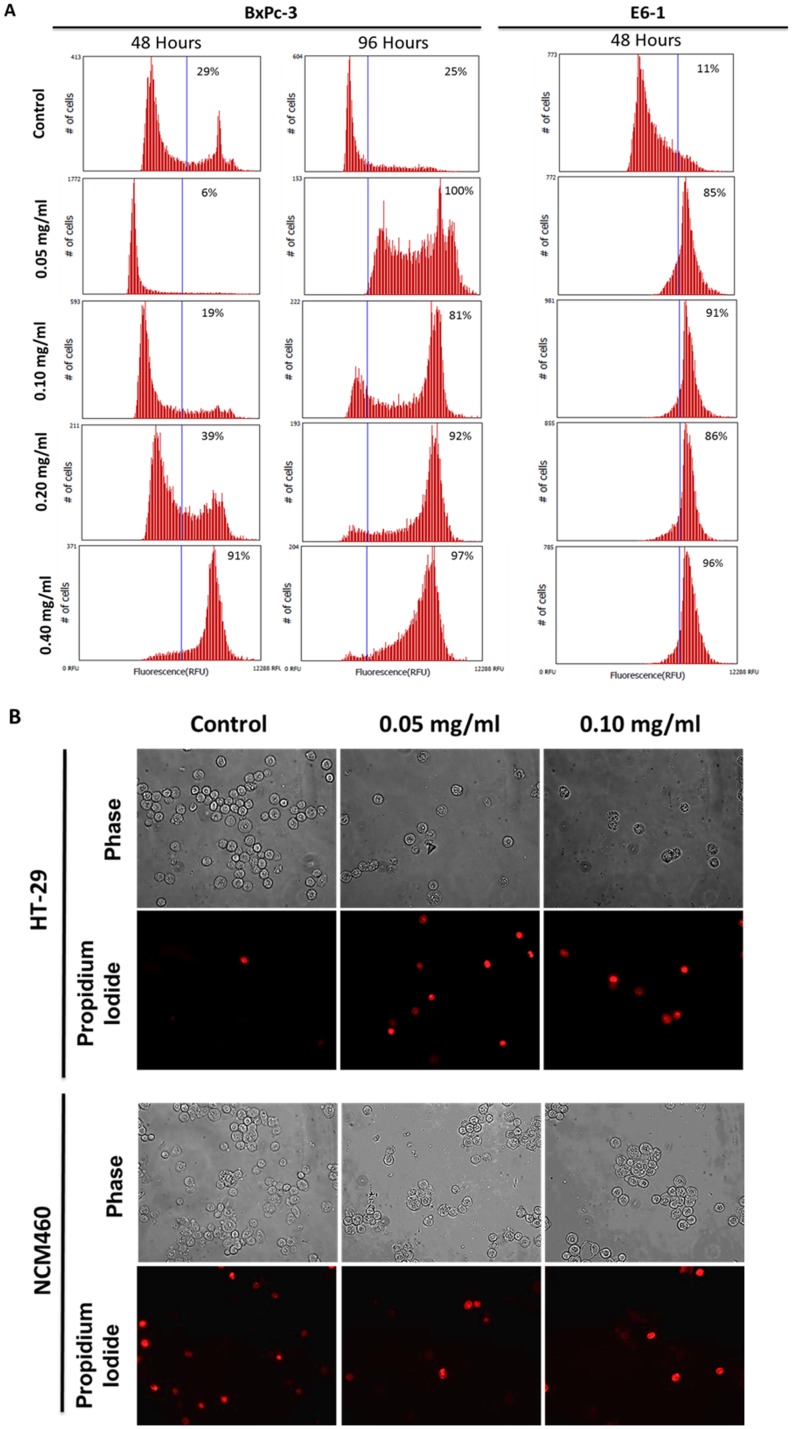
PLX Selectively Induces Cell Death in Human Cancer Cells in a Dose & Time Dependent Manner. (A) Following treatment of Human pancreatic (BxPc-3) cancer and T cell leukemia cells with PLX, at indicated time points, cells were incubated with propidium iodide and assessed for the induction of cell death by image-based cytometry. (B) Similar experiments were carried out in human colon cancer cells (HT-29) and normal colon epithelial cells (NCM460). Fluorescence microscopy was used to assess the induction of cell death as characterized by presence of propidium iodide positive cells. Images were taken at 400× magnification on a fluorescent microscope. Scale bar = 15 µm.

**Figure 3:**
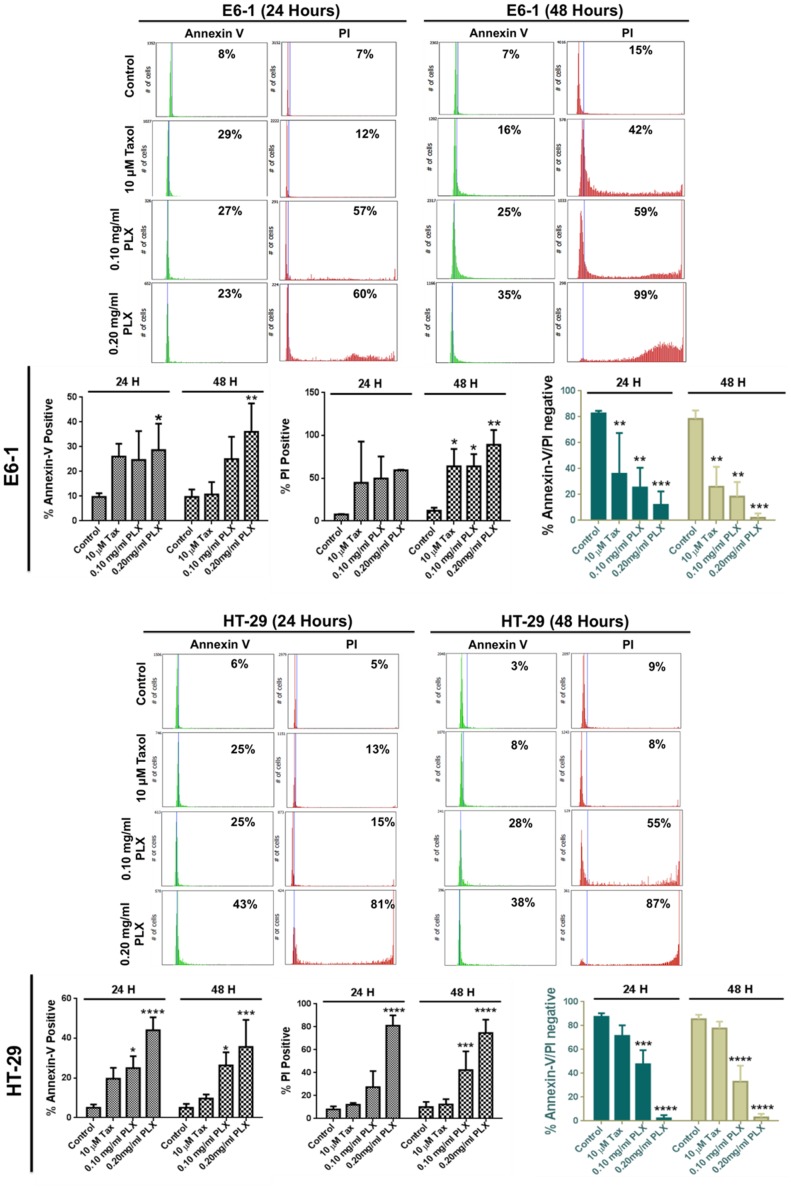
Quantification of Cell Death Induction Following PLX Treatment. Image-Based cytometry was used to quantify apoptotic induction (% Annexin V positive), followed by necrosis (% PI positive) in E6-1 and HT-29 cells following PLX treatment. The lack of annexin V or PI staining was used as an indication of live cells following treatment (%Annexin V/PI negative cells) (*P<0.05, ** P<0.003, ***P<0.0001). (E) To further confirm the induction of apoptosis.

The first step in understanding the effect of long pepper extract in this study was to assess the effect of PLX on the viability of cancer cells. Following treatment with increasing concentration of PLX at increasing time points, cells were incubated with a water soluble tetrazolium salt, which gets metabolized to a red formazan product by viable cells with active metabolism. This product can then be quantified by absorbance spectrometry. We observed the efficacy of crude PLX in reducing the viability of cancer cells, including colon (HCT116), pancreatic (BxPC-3), ovarian cancer (OVCAR-3) and melanoma cells. This effect was dose and time dependent (Figure 1). To further evaluate the anticancer activity of PLX, we wanted to assess its role in cell death and its selectivity to cancer cells. Our results demonstrate that PLX is able to selectively induce cell death in cancer cells (colon, pancreatic and leukemia) in a dose and time dependent manner, as characterized by the increase in propidium iodide positive cells in cancer cells treated with PLX (Figure 2). Furthermore, this effect was selective, as normal colon epithelial cells remained unaffected by this treatment, at the same concentrations and time-points (Figure 2B). These results were quantified using image-based cytometry to determine the percentage of cells undergoing apoptosis and total cell death. We observed a 30–40% increase in annexin V positive cells, following PLX treatment and an 80–100% PI positive increase in the same cell samples, confirming the induction of apoptosis, following by necrosis in cultured cancer cells (Figure 3).

**Figure 4:**
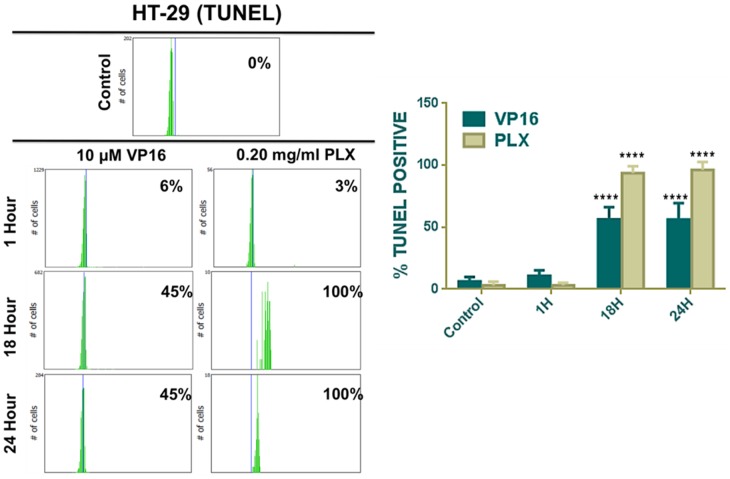
PLX Induces Double-stranded DNA Breaks in Cancer Cells. TUNEL labeling was used to detect DNA fragmentation. Following PLX and VP16 (as a positive control for DNA damage) treatment, cells were labelled with DNA staining solution and quantified by image-based cytometry. Treated cells were compared to the control untreated cell sample. (****P<0.0001).

DNA fragmentation is a key biochemical feature of apoptosis. To further confirm this induction of apoptosis, TUNEL labelling to detect DNA fragmentation was employed. Quantification results from image-based cytometry show the efficacy of PLX in inducing apoptosis, following DNA fragmentation in HT-29 colon cancer cells in a time dependent manner. VP16, a known chemotherapeutic agent with DNA damaging capabilities, was used as a positive control (Figure 4).

**Figure 5:**
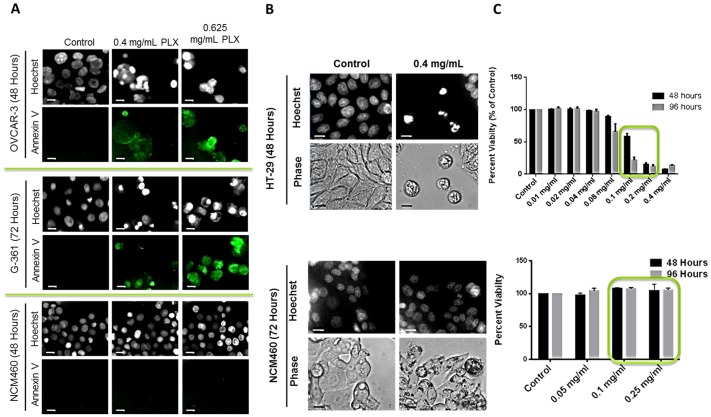
PLX Selectively Targets Cancer Cells for Apoptosis Induction. Subsequent to treatment with PLX, cells (Ovarian; OVCAR-3, Melanoma; G-361 and Normal Colon Epithelia cells (NCM460) were stained with Hoechst to characterize nuclear morphology and Annexin-V to detect apoptotic cells (A) and cellular morphology by phase contrast microscopy (B); Images were taken at 400× magnification on a fluorescent microscope. Scale bar = 15 µm. (C) Following PLX treatment, HT-29 colorectal cancer cells and non-cancerous NCM460 cells were incubated with WST-1 cell viability dye for 4 hours and absorbance was read at 450 nm and expressed as a percent of the control. Values are expressed as mean ± SD of 3 independent experiments. **P<0.0001.

Additionally, apoptosis induction in various cancer cells, melanoma (G-361), ovarian and colon cancer (HT-29) cells, was confirmed by Annexin-V binding assay. This induction of apoptosis was confirmed to be selective to cancer cells, as normal colon cells (NCM460) remained unaffected by PLX treatment. This was indicated by nuclear condensation, cell morphology and externalization of phosphatidyl serine to the outer leaflet of the cell membrane, as indicated by Hoechst staining, phase contrast images and binding of annexin V dye respectively (Figure 5A and B). The selectivity of PLX to cancer cells was further confirmed by the WST-1 cell viability assay that showed that PLX was highly effective at such low doses, a therapeutic window was easily observed (Figure 5C). Treatment of HT-29 with 0.20 mg/ml effectively reduced the viability by approximately 90%, while NCM460 cells remained at 100% viability at the same dose. This indicates that PLX can be more effective at very low doses, further reducing the chances of toxicity associated with treatment.

### PLX Induces Caspase-Independent Apoptosis in Human Cancer Cells

**Figure 6:**
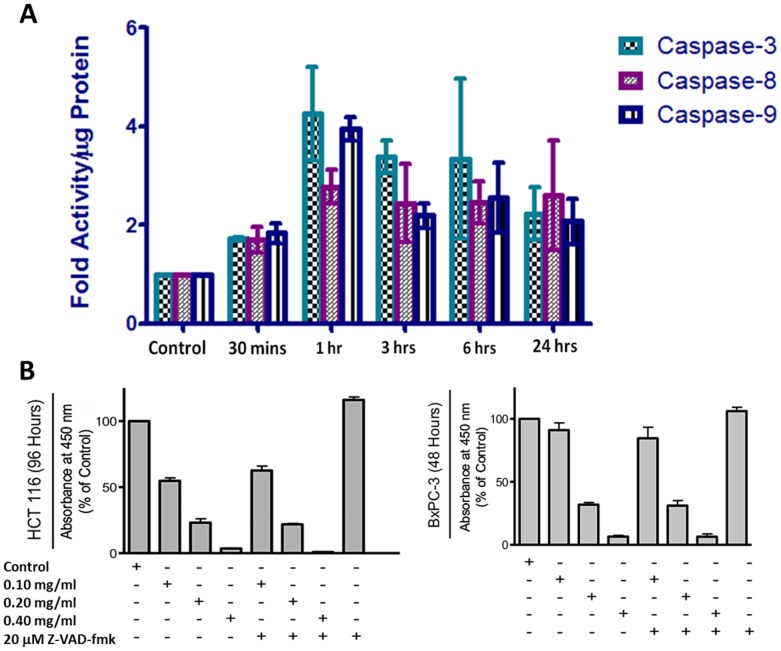
Long Pepper Extact (PLX) Activates the Extrinsic & Intrinsic Pathways of Apoptosis. Following treatment with 0.10 mg/ml PLX, at indicated time points, BxPc-3 cells were collected, washed and incubated with lysis buffer to obtain cell lysate. The cell lysate was incubated with caspase substrates, specific to each caspase (3, 8 and 9) and incubated for an hour. Fluorescence readings were obtained using a spectrofluorometer. An average of 6 readings per well and a minimum of three wells were run per experiment. The results here are reported as activity per µg of protein (in fold) and the average of three independent experiments is shown. (**B**) The reduction in viability was caspase independent, as a pan-caspase inhibitor, Z-VAD-fmk could not prevent the loss of viability induced by PLX treatment in colon and pancreatic cancer cells. Absorbance was read at 450 nm and expressed as a percent of the control. Values are expressed as mean ± SD from quadruplicates of 3 independent experiments. **P<0.0001.

Caspases are cysteine aspartic proteases that play a predominant role as death proteases [17]. Their roles in various cell death processes remains controversial, as their activation or inhibition could be essential to the progression of inhibition of cell death pathways [18], [19]. To assess the role of caspases in our study, following treatment with 0.10 mg/ml PLX, at indicated time points, BxPc-3 cells were collected, washed and incubated with lysis buffer to obtain cell lysate. The cell lysate was incubated with caspase substrates, specific to each caspase (3, 8 and 9) and incubated for an hour. Fluorescence readings were obtained using a spectrofluorometer. Our results indicate that PLX is able to activate both pathways (extrinsic and intrinsic apoptosis) in a time dependent manner. This was observed as rapid activation of caspases-3, 8 and 9 were observed as early as an hour, following treatment (Figure 6A).

To determine the importance of these activated caspases to the apoptosis-inducing effect of PLX, colon (HCT116) and pancreatic (BxPc-3) cancer cells were pre-treated with a pan-caspase inhibitor, Z-VAD-fmk (20 µM), for an hour before treatment with PLX. Following treatments, the WST-1 cell viability assay was used to assess for viability and efficacy of PLX. Our results indicate that the inhibition of caspases could not prevent the reduction of viability (Figure 6B), signifying that the effect of PLX in cancer cells is independent of caspase activation.

### Long Pepper Extract Induces Oxidative Stress and Targets the Mitochondria of Cancer Cells

**Figure 7:**
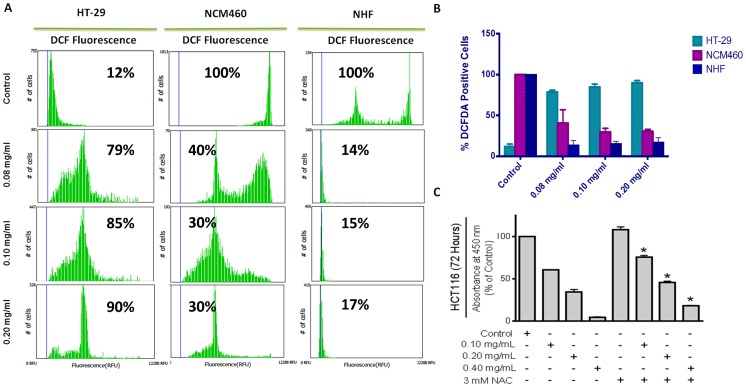
PLX Causes but is Not Dependent on the Production of Reactive Oxygen Species (ROS). (A) Colon cancer (HT-29), Normal Colon Epithelial (NCM460) and Normal Human Fibroblast (NHF) cells were treated with PLX for 48 hours, following which, they were incubated with H_2_DCFDA and fluorescence results were obtained using an image based cytometer. Results were quantified using Graphpad prism 6.0 (B). (C) HCT116 colon cancer cells were treated with 3 mM N-acetylcysteine for an hour prior to PLX treatment. Cells were then treated PLX at indicated concentrations for 72 hours, following which the WST-1 assay was performed. Absorbance readings were taken at 450 nm and expressed as a percent of the control. Values are expressed as mean ± SD from quadruplicates of 3 independent experiments. *p<0.05.

Generation of oxidative stress has been well established as a major player in the induction of several cell death processes, especially apoptosis [20], [21]. The next part of our study focused on the role of oxidative stress in PLX induced apoptosis. Following treatment with PLX for 48 hours, cells were incubated with 2′,7′-Dichlorofluorescin diacetate H_2_DCFDA for 45 minutes. The resulting green fluorescence histograms were obtained using a TALI image-based cytometer. From the results, it was observed that PLX induced extensive generation of whole cell reactive oxygen species (ROS) in HT-29 colon cancer cells, while acting to suppress any ROS present in the non-cancerous cell lines, NCM460 and normal human fibroblasts (NHF) (Figure 7A & B). This confirms our results of selectivity and indicates that PLX might act as a pro-oxidant in cancer cells in order to induce apoptosis.

To determine if this oxidative stress was essential to PLX activity, HCT116 colon cancer cells were pre-treated with N-acetyl-L-cysteine (NAC), a well-established anti-oxidant, used extensively in vitro studies [22], [23], before treatment with PLX. Subsequent to PLX treatment, cells were analyzed for effect of PLX on viability, using the WST-1 viability assay. The results suggest that although PLX acts to induce oxidative stress to cause apoptosis, this oxidative stress is not essential to its activity. Both the cells treated with PLX alone and NAC followed by PLX showed a reduction in their viability (Figure 7C).

The mitochondria have also been shown to play a major role in the progression and execution of apoptosis. The permeabilization of the mitochondrial membrane usually leads to the release of pro-apoptotic factors, including cytochrome c, apoptosis inducing factor (AIF) and endonuclease G (EndoG) [5], [24]. These factors cause a caspase-independent pathway for apoptosis to pass through and could bypass the antioxidant effects of NAC observed in figure 7C.

**Figure 8:**
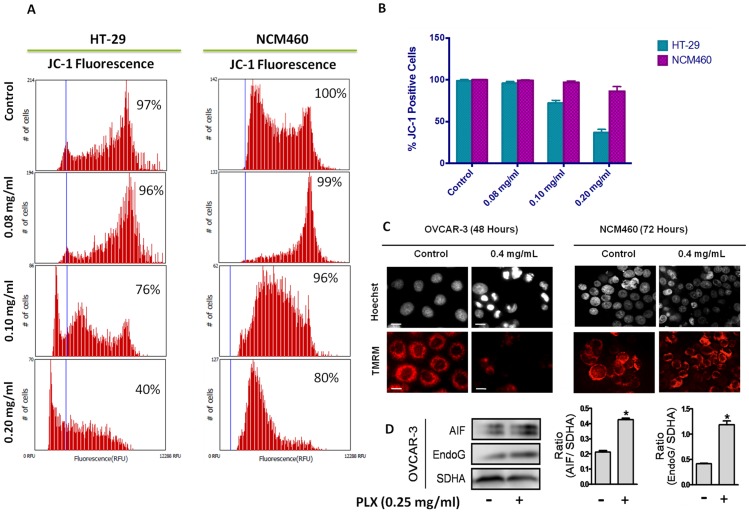
PLX Destabilizes the Mitochondrial Membrane of Cancer Cells. Colon cancer (HT-29), Ovarian cancer (OVCAR-3) and Normal Colon Epithelial (NCM460) cells were treated for 48 hours with PLX, following which, they were incubated with JC-1 (A) or TMRM (C) cationic mitochondrial membrane permeable dyes. Fluorescence readings were obtained using image based cytometry (A) and fluorescence microscopy; corresponding Hoechst dye images are also shown (C). Images were taken at 400× magnification on a fluorescent microscope. Scale bar = 15 µm. (D) Isolated mitochondria of OVCAR-3 cells were treated directly with PLX or solvent control (ethanol) for 2 hours. Following treatment, samples were centrifuged, to obtain mitochondrial supernatants, which were examined for the release of pro-apoptotic factors, AIF and EndoG via western blot analyses, and mitochondrial pellets which were probed for SDHA to serve as loading controls. Image is representative of 3 independent experiments demonstrating similar trends. Values are expressed as mean ± SD of quadruplicates of 1 independent experiment; *p<0.01 versus solvent control (ethanol).

To assess the efficacy of PLX on the mitochondria of cancer cells, OVCAR-3, HT-29 and NCM460 cells were stained with TMRM, a cationic dye that accumulates in healthy mitochondria. Mitochondrial membrane potential (MMP) dissipation was only observed in OVCAR-3 and HT-29 cells as seen with the dissipation of red TMRM fluorescence, by fluorescence microscopy and image-based cytometry (Figure 8A, B & C). Following mitochondrial membrane collapse, we wanted to determine if there was release of some pro-apoptotic factors. Western blot analysis was used to monitor for the release of AIF and EndoG from isolated OVCAR-3 mitochondria. Results demonstrate that PLX directly caused the release of both AIF and EndoG from the mitochondria of OVCAR-3 cells (Figure 8D). These results provide an insight to the mechanism of PLX action, where the mitochondria appear to be a direct target of PLX for the reduction of viability and the induction of apoptosis.

### Long Pepper Extract is Well-Tolerated in Animal Models

**Figure 9:**
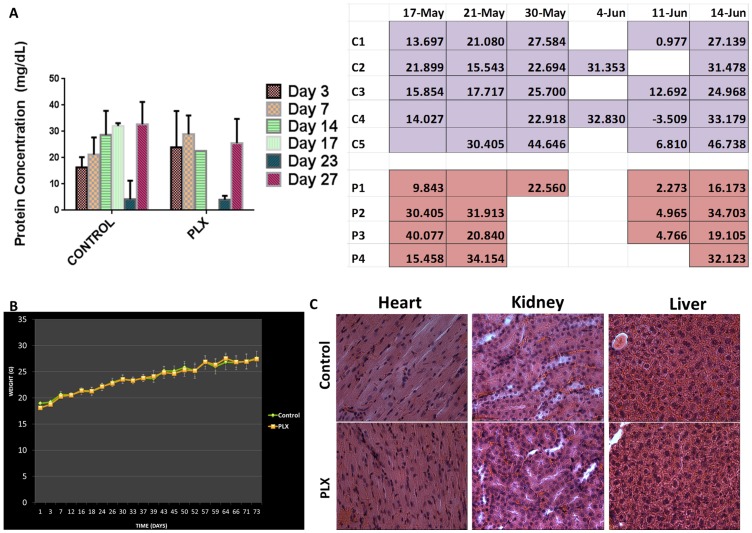
PLX is Well-Tolerated in Mice Models. Balb/C mice were divided into three groups (3 animals/control (untreated), 3 animals/gavage control (vehicle treatment) and 4 animals/treatment group). The control untreated group was given plain filtered water, while the second and third group was given 50 mg/kg/day vehicle (DMSO) or PLX, respectively. Mice were assessed for toxicity with protein urinalysis by Bradford Assay and dipstick analysis (A) and weight changes (B). (C) Hematoxylin and Eosin stained tissue sections of the liver, heart and kidney of control versus PLX treated group. Images were obtained on a bright field microscope at 63× objective.

Long pepper extracts (mainly water extracts) have been used for centuries and have been associated with various benefits [9]. With all these anecdotal reports of benefits, there have been no reports of toxicities associated with its use. To further scientifically evaluate and validate the safety of PLX, balb/c mice were orally gavaged with 50 mg/kg/day vehicle (DMSO) or PLX for 75 days and the mice were observed for signs of toxicity. To assess for toxicity, mice were weighed twice a week, urine was collected for protein urinalysis studies and following period of treatment, mice were sacrificed and their organs were obtained for pathological analysis by a certified pathologist at the University of Guelph (Dr. Brooke). Results from this part of the study demonstrate that there was no weight loss overall in mice that were given PLX supplemented water (Figure 9).

To further assess toxicity, urine was collected from mice once a week and protein urinalysis was performed using a urine dipstick and a Bradford protein concentration assay. Protein urinalysis results indicate that there were trace amounts of protein in the urine of mice both from the control and the PLX group, with trace readings corresponding to protein concentrations between 5 and 20 mg/dL. Bradford assays confirm the results obtained by dipstick urinalysis (Figure 9A). There was no major difference between the control group and PLX group, confirming the lack of toxicity associated with oral administration of PLX in drinking water. Furthermore, the hearts, livers and kidneys were obtained following the toxicity study, sliced and stained with hematoxylin and eosin. Results show no gross morphologic difference between the control and the treatment group, confirming the lack of toxicity associated with PLX treatment. Results from the pathologist, indicate that the presence of any lesions in the tissues are minimal or mild and interpreted as either background or incidental lesions and the lack of lesion type and frequency was enough to conclude no toxicological effect of PLX to the balb/c mice (Table 2).

**Table 2:** Summary of Histological Lesions in Balb/C Mice on PLX regimen.

	No Treatment		Vehicle (Gavage Control)			Long Pepper Extract (Treatment group)			
	M1	M2	M1	M2	M3	M1	M2	M3	M4
**Liver:**									
-Infiltration, leukocyte, predominantly mononuclear, minimal		**X**	**X**		**X**	**X**			**X**
-Focal mineralization, minimal									
-Hepatocyte necrosis, minimal									**X**
-Focus of cellular alteration, eosinophilic, minimal			**X**	**X**				**X**	
-Hepatocyte vacuolation, lipid type, minimal			**X**	**X**			**X**		
- Hepatocyte vacuolation, lipid type, mild	**X**			**X**				**X**	**X**
Fibrin thrombus			**X**						
**Heart:**									
-Infiltration, leukocyte, predominantly mononuclear, minimal		**X**				**X**			**X**
Myofiber separation and vaculation, minimal (suspect artifact)		**X**	**X**						**X**
**Kidney:**									
- Infiltration, leukocyte, predominantly mononuclear, minimal	**X**	**X**		**X**		**X**		**X**	
Tubule vacuolation, minimal					**X**				**X**
Fibrin or other extracellular matrix, glomerulus								**X**	

### Oral Administration of Long Pepper Extract Halts the Growth of Human Colon Cancer Xenografts in Immunocompromised Mice

**Figure 10:**
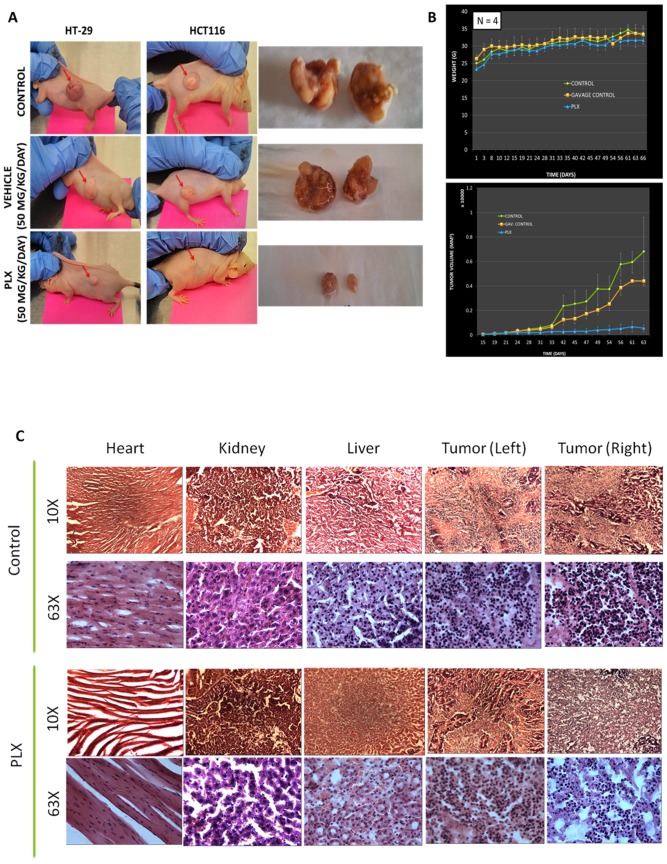
PLX Halts Growth of Colon Tumors in Xenograft Models. CD-1 nu/nu mice were subcutaneously injected with colon cancer cells; HT-29 (p53^−/−^) on the left flank and HCT116 (p53^+/+^) on the right flank. (A) Representative tumor size control mice and 50 mg/kg/day vehicle or PLX treated mice, respectively. PLX halted the growth of both HT-29 and HCT116 tumors *in-vivo*. (B) Average body weights of control and PLX treated mice. The body weights did not vary significantly during the study. Tumor volumes were measured and tumor curve shows the efficacy of 50 mg/kg/day oral administration of PLX. (C) Histopathological analysis of tissue samples obtained from control and PLX-treated animals. Hematoxylin and Eosin stained tissue sections of the livers, hearts, kidneys and tumors. Images were obtained on a bright field microscope at 10× and 63× objective.

Following efficacy studies, we wanted to further study the efficacy of PLX. For this study, CD-1nu/nu immunocompromised mice were subcutaneously injected with HT-29 cells (left) and HCT116 cells (right). Following the establishment of tumors, mice were separated into three groups, a control group, a vehicle (Me_2_SO) group and a PLX treated group. Mice were observed for 75 days, with weights and tumor volumes measured twice a week. Results demonstrate that oral administration of PLX could suppress the growth of both p53 WT (HCT116) and p53 mutant (HT-29) tumors *in-vivo*. There were no signs of toxicity, as indicated by increasing weights during the study (Figure 10A & B). Furthermore, H & E staining revealed less nuclei in the PLX treated group, compared to the control group, however, as observed in the toxicity studies, there were no gross morphological differences in the livers, kidneys and hearts of the control and PLX groups (Figure 10C).

### Analysis of Long Pepper Extract

**Figure 11:**
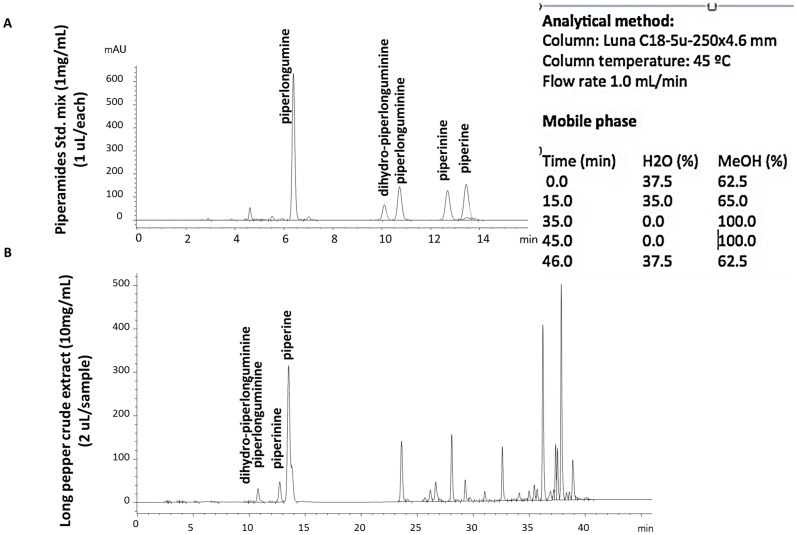
HPLC Analysis of PLX. Chromatograms of *Piper longum* extract (PLX) used for this study (10 mg/mL at 2 µL/Sample) (B) compared to Piperamides Standard mix (1 mg/mL at 1 µL/standard) (A).

The availability of several species of long pepper and the host of compounds present within them make it essential to characterize the long pepper extract that has shown potent anticancer activity, both in *in-vitro* and *in-vivo* studies. We ran an HPLC profile study on the crude ethanolic extracts, compared with a piperamide standard mix. The chromatogram profile show that our PLX extract contained several classes of compounds known to be present in piper species, including piperines, piperlongumine and dihydropiperlongumine (Figure 11A & B), suggesting that our extract is a member of the *Piper longum* species.

## Discussion

In this report we demonstrate for the first time, the selective anticancer potential of an ethanolic extract of the fruit of long pepper (PLX) in several cancer cell lines. PLX effectively reduced the viability of cancer cells and induced apoptosis in a dose- and time-dependent manner, at low doses, allowing for a greater therapeutic window in *in-vitro* studies (Figure 1–5). This apoptosis inducing effect was found to be independent of caspases, cysteine aspartic proteases that play a role in the progression and execution of apoptosis (Figure 6B). These results suggest that PLX is not toxic to non-cancerous cells at such low doses, as was observed in the cancer cells. Selectivity and lack of toxicity was confirmed with *in-vivo* toxicological studies.

Damage to the kidneys is a common occurrence during various types to toxic therapies. This damage to the kidney results in large amounts of protein (>3.5 g/day) leaking into the urine [25], [26], and this can be measured by various assays. Lack of toxicity was confirmed by the lack of increased protein concentration in the urine samples collected from both the control group and PLX treated group, by two different assays. The urine dipstick method indicated that all urine samples from the control and PLX groups had trace amounts of protein, corresponding to concentrations between 5 mg/dL and 20 mg/dL, well within the acceptable concentration range. Bradford protein assay showed a concentration of approximately 30 mg/dL most days urine was collected (Figure 9A). This is still within the acceptable range of protein concentration in urine. These results confirm anecdotal studies that suggest no associated toxicity or side effects observed with take long pepper extracts. The efficacy of PLX in *in-vivo* models also showed that not only was PLX well-tolerated, it was also effective at halting the growth of human tumor xenografts of colon cancer in nude mice (Figure 9A and B).

The next step in understanding the effect of PLX on cell death induction in cancer cells was to identify the mechanism of apoptosis induction observed following PLX treatment. The role of oxidative stress in cell death processes has been well characterized. It is well established the reactive oxygen species (ROS) could be the cause or effect of apoptosis induction in cells [20]. Some studies have suggested cancer cells to be more dependent on cellular response mechanisms against oxidative stress and have exploited this feature to selectively target cancer cells [10]. The role of ROS generation in PLX-induced apoptosis was assessed following treatment. In this study, we found that PLX induced whole cell ROS production in a dose dependent manner, as indicated by the increase in green fluorescence of H_2_DCFDA dye, cleaved by intracellular esterases and oxidized by ROS present (Figure 7A & B). However, we observed that ROS generation was not completely essential to PLX activity, as the presence of N-acetylcysteine could not entirely hamper the ability of PLX to reduce the viability of colon cancer cells (Figure 7C).

The caspase-independence observed in figure 6B, suggest that PLX is acting through pro-apoptotic factors other than caspases. The mitochondria play a major role in the progression and execution of apoptosis. The permeabilization of the mitochondrial membrane usually leads to the release of pro-apoptotic factors, including cytochrome c, apoptosis inducing factor (AIF) and endonuclease G (EndoG) [5], [17]. AIF and EndoG execute apoptosis in a caspase-independent possibly leading to the caspase- and partial ROS-independence observed. We show here that PLX caused MMP dissipation in cancer cells, while non-cancerous NCM460 cell mitochondria remained intact following treatment (Figure 8A–C). The dissipation of the mitochondrial membrane led to the release of AIF and EndoG (Figure 8D), allowing for the progression and execution of apoptosis in the absence of caspases and oxidative stress, providing insight to the mechanism of PLX action in cancer cells. Cancer cells differ from non-cancerous cells in variety of ways, which could enhance the selectivity of PLX to cancer cells. The Warburg effect is characterized by the high dependence of cancer cells on glycolysis and low dependence on mitochondria for energy production in cancer cells, therefore creating a more vulnerable target in cancer cell mitochondria [27]. Moreover, various anti-apoptotic proteins associated to the mitochondria have been reported to be highly expressed in cancer cells. Such proteins could serve as targets for selective cancer [28]–[30].

Unlike isolated natural compounds, there are usually more benefits to using a whole plant extract, with multiple pharmacologically active phytochemicals, than a single isolated compound. Multiple components within extracts could have many different intracellular targets, which may act in a synergistic way to enhance specific activities (including anticancer activities), while inhibiting any toxic effects of one compound alone. Additionally, the presence of multiple components may possibly decrease the chances of developing chemoresistance [31]. Moreover, natural extracts can be administered orally to patients, as a safe mode of administration. Some known compounds of the long pepper plants have been isolated and studied for their various activities [9]–[14].

We now report that the botanical identification of long pepper that was used for this study is *Piper longum* L. (Piperaceae), obtained from India. The phytochemical analysis of our material confirmed that this is the *Piper longum* species. We used the extract of the fruit in this study, since it is the usual part used medicinally and can be harvested sustainably. Although the fruit contains related piperamides, it does not contain piperlongumine, which is mainly present in the root of this plant [32]. The other piperamides present in the fruit such as dihydropiperlongumine likely have similar bioactivity. The small peak of piperlongumine observed in the HPLC chromatogram in Figure 11, as piperlongumine may be due to the reduction of piperlongumine to the larger dihyropiperlongumine peak that we observe. The analysis is consistent with *P. longum* fruits, and this is very important and points to the novel findings regarding the anticancer activity of this composition of components.

In a previous study that showed the efficacy of piperlongumine, high concentrations of 10 µM were required for significant cell death induction in cancer cells [10]. In this present study, we report the use of low concentrations of the complex mixture of the ethanolic long pepper extract (containing many bioactive and pharmacologically active compounds) was sufficient in inducing apoptosis in cancer cells selectively. This indicates that the individual bioactive compounds (present in sub micromolar concentrations within the extract) could act synergistically to induce apoptosis in cancer cells at very low concentrations, unlike a single identified compound. These findings highlights that the *Piper spp.* contain novel compounds with potent anticancer activity, in addition to piperlongumine.

In conclusion, our results demonstrate that long pepper extract (PLX), with a long historical use in traditional medicine, is selective in inducing apoptotic cell death in cancer cells by targeting non-genomic targets (e.g. the mitochondria). It is well tolerated in mice models and effective in reducing the growth of human tumor xenotransplants in animal models, when delivered orally. This could open a window of opportunity to develop a novel, safer cancer treatment, using complex natural health products from the Long Pepper.
